# Deep brain stimulation for obsessive-compulsive disorder : A look back over 24 years of use

**DOI:** 10.1192/j.eurpsy.2025.2124

**Published:** 2025-08-26

**Authors:** A. Karmous, H. Ghabi, A. Hajri, E. Khelifa, A. Maamri, H. Zalila

**Affiliations:** 1 Emergency and outpatient department; 2 Department G, Razi hospital, Manouba, Tunisia

## Abstract

**Introduction:**

Deep brain stimulation (DBS) was first used in 1999 for the treatment of resistant obsessive-compulsive disorder (OCD), and it was not until 2009 that the Food and Drug Administration approved it for this purpose.

**Objectives:**

The aim of our study was to investigate the safety and efficacy of DBS through a systematic review.

**Methods:**

A systematic review was conducted. PubMed via Medline, Google Scholar and Semantic Scholar were used as search engines. The keywords used were (“Obsessive compulsive disorder” or “OCD”) and (“Deep brain stimulation” or “DBS”). Clinical trials and observational studies assessing the efficacy of DBS for OCD, published from inception to December 2023 and written in English or Frensh were analysed. The inclusion criteria were a main diagnosis of OCD, DBS conducted for therapeutic purposes and a response to DBS assessed by the Yale-Brown Obsessive-Compulsive scale (Y-BOCS).

**Results:**

Thirty four studies were included in the final analysis with a reported total of 316 cases. In 58.2% of cases, patients were female. The average age at the time of surgery of implanting the stimulation device was 40.9 ± 7.8 years. The mean time from onset of symptoms to surgery was 22.4 ± 4.6 years. The mean initial Y-BOCS score was 33 ± 3.7. The mean response rate, defined as a reduction in Y-BOCS score of more than 35 % was 70.7% ± 24.8. The maximum symptom reduction was reached between 12 to 14 months after implantation in most studies. Hypomania was the most frequent side effect reaching 45% in some studies. Intracranial hemorrhage secondary to surgery was the most serious complication and did not exceed 5 % in any study. No clear predictive factors for response to DBS were identified.

**Conclusions:**

DBS appears to be a promising therapy for patients with resistant OCD. This innovative approach, combined with ongoing advancements in neurotechnology, offers hope for the future of OCD treatment. However, no predictors of response have yet been established.

**Disclosure of Interest:**

None Declared

